# Primary Care Continuity, Frequency, and Regularity Associated With Medicare Savings

**DOI:** 10.1001/jamanetworkopen.2023.29991

**Published:** 2023-08-21

**Authors:** Dilara Sonmez, George Weyer, Daniel Adelman

**Affiliations:** 1 University of Chicago Booth School of Business, Chicago, Illinois; 2 University of Chicago Medicine, Chicago, Illinois

## Abstract

**Importance:**

Reducing Medicare expenditures is a key objective of Medicare’s transition to value-based reimbursement models. Improving access to primary care is an important way to reduce expenditures, yet less is known about how visits should be organized to maximize savings.

**Objective:**

To examine the association between Medicare savings and primary care visit patterns.

**Design, Setting, and Participants:**

This retrospective cohort study used data from a 5% sample of traditional Medicare claims from 2016 to 2019. Participants had at least 3 primary care visits with at least 180 days between the first and the last visit, were not enrolled in Medicare Advantage, did not have end-stage kidney disease, and were not institutionalized. Data were analyzed from June 2022 to April 2023.

**Exposures:**

Primary care visit patterns: visit frequency, regularity, continuity of care.

**Main Outcomes and Measures:**

Savings in Medicare expenditures; risk-adjusted Medicare expenditures, number of emergency department (ED) visits, and hospitalizations.

**Results:**

Among 504 471 beneficiaries (298 422 [59.16%] women; mean [SD] age, 74.26 [10.41] years), temporally regular visits with higher continuity were associated with the highest savings. For these patients, the savings increased with increasing visit frequencies, with peak savings observed at higher visit frequencies as clinical complexity increased. As regularity and continuity decreased, the association between savings and visit frequencies progressively inverted. The group with a regular and highly continuous pattern was associated with greater savings (175.87%; 95% CI, 167.40% to 184.33%; *P* < .001), lower risk-adjusted expenditures (−16.61%; 95% CI, –16.73% to –16.48%; *P* < .001), fewer risk-adjusted ED visits (−40.49%; 95% CI, –40.55% to −40.43%; *P* < .001), and fewer risk-adjusted hospitalizations (−53.32%; 95% CI, –53.49% to –53.14%; *P* < .001) compared with the irregular noncontinuous group.

**Conclusions and Relevance:**

In this cohort study, savings in Medicare expenditures and improvements in acute care utilization were associated with visit frequency, regularity, and continuity in primary care in an interrelated fashion such that optimization of primary care visit patterns along each axis were associated with the largest improvement in outcomes. Demonstrating the magnitude and interdependence of these associations is useful for health care professionals and policymakers as Medicare continues its transition to value-based reimbursement models.

## Key Points

**Question:**

What is the association of primary care visit patterns with outcomes?

**Findings:**

In this cohort study with 504 471 continuously enrolled Medicare fee-for-service beneficiaries who had at least 3 primary care visits from 2016 to 2018, having regularly scheduled visits to the same primary care clinician was associated with higher savings. The greatest savings were associated with higher frequencies as patient complexity increased.

**Meaning:**

These findings suggest that having regular primary care visits with the same clinician is strongly associated with Medicare savings, an association that is optimized at greater visit frequencies for patients of higher complexity.

## Teaser

This cohort study examines data for Medicare fee-for-service beneficiaries to determine the association between savings in Medicare expenditures and the frequency, regularity, and continuity of care in primary care visits.

## Introduction

Medicare has announced a strategic goal of having all traditional fee-for-service beneficiaries in an accountable care relationship by 2030.^1^ Primary care is foundational within accountable care models both because patients are attributed to accountable care organizations through primary care relationships and also because primary care represents an accessible, low-cost,^2^ and comprehensive point for health care access that has been broadly associated with higher quality, lower health care costs, and longer life expectancy.^3^

Primary care practice patterns are increasingly recognized as important factors mediating the association of primary care to outcomes, including health care savings. For example, greater continuity with both primary care clinicians and practices has been associated with lower costs of care, reduced acute care utilization, and improved population-level mortality.^4,5,6^ Recent work by Rose et al^7^ identified an association between the temporal regularity and frequency of primary care visits and lower health care costs.

Patients who access primary care in a discontinuous and irregular fashion are more likely receiving reactive care, with visits occurring with an available health care professional after a need develops. In comparison, patients receiving regular visits with a continuity clinician are more likely receiving proactive care. These patient relationships may also be associated with greater trust or stronger physician-patient relationship that might lead to better outcomes.^8^ We hypothesized that the former type of care will be associated with greater costs and acute care utilization than the latter.

## Methods

### Study Population

In this retrospective cross-sectional cohort study, we used claims data from a nationally representative 5% sample of traditional Medicare beneficiaries from 2016 to 2019 to identify a cohort of continuously enrolled beneficiaries who had received at least 3 primary care visits from 2016 to 2018. Beneficiaries were initially selected for continuous enrollment in Medicare Parts A and B, excluding those with any period of enrollment in Medicare Advantage or with end-stage kidney disease, with a requirement that this period of continuous enrollment include the outcome year (2019) as well as at least 1 year but up to 3 years of the baseline period (2016-2018). We required beneficiaries to be alive through the end of 2019. A total of 1 077 676 patients satisfied these enrollment criteria.

We restricted this sample to beneficiaries with a minimum of 3 primary care visits documented during the baseline years and required that at least 180 days separate the first and last primary care visit. *Current Procedural Terminology* codes used to identify primary care visits are listed in eTable 1 in Supplement 1. We excluded patients who were institutionalized during the study time period. Additional details about the exclusion criteria are provided in the eMethods and eFigure 1 in Supplement 2.

This study was reviewed by the University of Chicago institutional review board and determined to not be human participant research. We followed the Strengthening the Reporting of Observational Studies in Epidemiology (STROBE) reporting guidelines. Race and ethnicity data were obtained from the Medicare Master Beneficiary Summary File, which itself originates from Social Security Administration records.

### Variables

Primary care visit patterns were measured in the first 3 years, 2016-2018. The outcome variables were measured in the follow-up year, 2019. Primary care visit patterns were measured along 3 dimensions. The first measure was visit frequency, ie, the mean annual number of primary care visits. Second, we measured regularity of care, defined as the variability in the number of days between visits. Finally, we created a novel, time-weighted, continuity-of-care measure to assess the extent to which patients obtained their care from their most responsible primary care clinician or organization instead of visiting other primary care clinicians or organizations.

The primary outcome was savings in Medicare expenditures. Secondary outcomes were risk-adjusted values for Medicare expenditures, emergency department (ED) visits, and hospitalizations. We created 6 comparison groups by dividing beneficiaries into 2 groups based on their regularity values and then dividing each regularity group into 3 continuity subgroups. The regular, highly continuous group is referred to as *proactive* and its irregular, noncontinuous counterpart as *reactive*. We then reported the association between visit frequency and the primary and secondary outcomes for each of these 6 groups.

#### Frequency

The frequency of visits was measured by the annual number of primary care visits. The total number of visits during the years the beneficiary was eligible was divided by the total years the beneficiary was eligible and excluded any time spent hospitalized.

#### Regularity of Care

The coefficient of variation (CoV) of the time between successive primary care visits was calculated for each beneficiary as the mean number of days between successive primary care visits divided by its standard deviation. Beneficiaries were labeled as regular or irregular beneficiaries depending on their CoV values. A beneficiary with a CoV value below the 30th quantile CoV value of the sample was considered to have had regular primary care visits; for any other CoV value, the beneficiary was considered to have had irregular primary care visits.

#### Continuity of Care

A novel continuity-of-care measure was developed to test the hypothesis that spending a larger portion of time under the care of a single primary care clinician significantly affects health outcomes. In the medical literature, continuity of care is defined as the extent to which patients obtain primary care visits from their most responsible clinician as opposed to visiting other clinicians. High-quality primary care is defined as a sustained relationship that exists both at visits but also in between visits.^3,9^ We define a novel, time-weighted measure of continuity calculated using the normalized Shannon entropy of the time between visits to different clinicians. A beneficiary is considered to be under a clinician’s care from the visit date to that clinician until the visit date to another clinician. The share of time under different clinicians’ care constitutes a probability distribution for a beneficiary. Let *p_i_* be the share of a clinician *i*, and let *t* be a total number of days the beneficiary had been eligible. The entropy of this probability distribution is calculated as follows: −∑*_i_p_i_* / log*p_i_*.

A distribution closer to a uniform distribution has a higher entropy value. Because the entropy values of beneficiaries with different duration of eligibility were not comparable, normalized entropy values were calculated by dividing the entropy value of the beneficiary by log*t*. We call this metric the physician entropy. We also calculated organizational entropy, defining *p_i_* to be the share of a billing clinician *i*.

We defined continuity of care as a 2-dimensional variable, consisting of physician and organizational entropy. A beneficiary with the sum of physician and organizational entropy less than the 30th quantile, q_30_, was considered to be a highly continuous beneficiary. They corresponded to beneficiaries below the y = −x + q_30_ line, where the y-axis is the organizational entropy and the x-axis is the physician entropy. A visualization of how the cutoffs are applied is given in eFigure 2 in Supplement 2.

If a beneficiary has both a physician and organizational entropy value below the 65th quantile and above the 30th quantile, that beneficiary is considered to be moderately continuous. The rest of the beneficiaries are noncontinuous. The 2-dimensional structure of the continuity metric weakens the dependence of the continuity metric on visit frequency because beneficiaries with higher frequencies tend to have lower organizational entropies. It also gives credit for continuous care within the same physician group, even when the providing physician differs.

#### Savings in Medicare Expenditures

Medicare expenditures were calculated as the sum of Medicare expenditures documented in the inpatient, outpatient, skilled nursing facility, home health, hospice, durable medical equipment, and carrier claim files in 2019. Savings in Medicare expenditures, the primary outcome measure, were defined as the difference in the expected Medicare expenditures, which were risk adjusted, and the observed Medicare expenditure.

Risk adjustment was performed using the hierarchical condition categories (CMS-HCC) risk-adjustment model published by the Centers for Medicare and & Medicaid Services.^10,11,12^ This model assigns a risk adjustment factor (RAF) score to specified diagnosis codes and adds additional risk adjustment weight for demographic information, including sex, age, reason for Medicare eligibility, and institutional status.^11^

#### Risk-Adjusted Medicare Expenditures

The ratio of total observed Medicare expenditures to the total expected Medicare expenditures in a regularity-continuity subgroup was multiplied by the population average of the observed Medicare expenditures for risk adjustment. To find the expected Medicare expenditure of a beneficiary, we used a gamma generalized linear model with county fixed effects (GLME). In the GLME model, demographic characteristics (sex, age, race, duality status, reason for Medicare eligibility, county) and the HCC comorbidities of patients were used as regressors.

#### Risk-Adjusted Number of ED Visits

ED visits that did not result in an inpatient stay were counted using the outpatient claims. A Poisson GLME model with county fixed effects was fit to find the expected number of ED visits of beneficiaries, and the same method of risk adjustment with risk-adjusted Medicare expenditures was used.

#### Risk-Adjusted Number of Hospitalizations

Inpatient stays in traditional acute care hospitals or critical access hospitals were counted. A Poisson GLME model with county fixed effects was fit to find the expected number of hospitalizations of beneficiaries in the cohort. The same method of risk adjustment with risk-adjusted Medicare expenditures was used.

### Statistical Analysis

**Figure 1.**
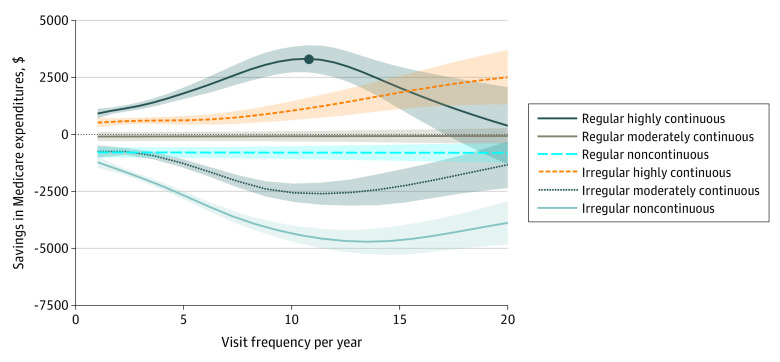
Savings in Medicare Expenditures vs Frequency by Regularity and Continuity Subgroups Association between savings in Medicare expenditures and frequency. The curves correspond to different regularity and continuity subgroups for comparison. Shading indicates 95% confidence intervals.

**Figure 2.**
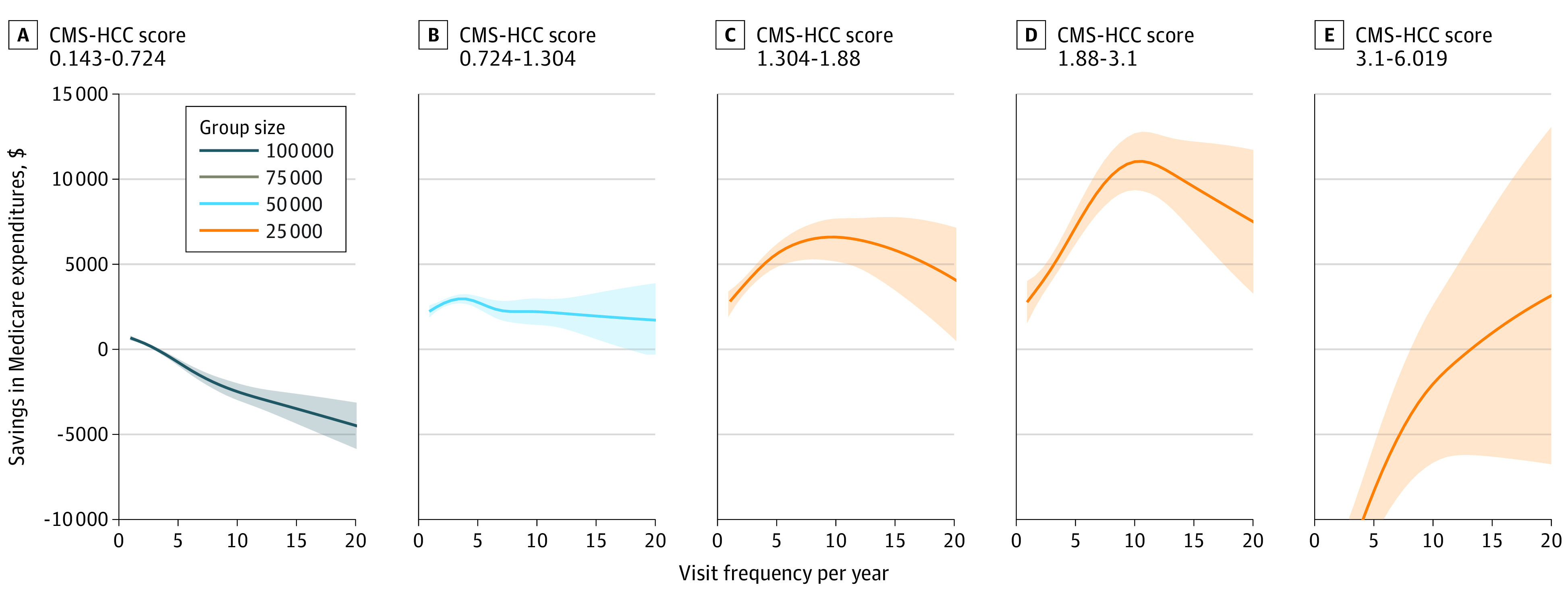
Proactive Group Savings in Medicare Expenditures vs Frequency Stratified by Risk Adjustment Factor An increasing number of optimal visit frequencies is found for beneficiaries with increasing risk scores when the proactive group is further divided into risk-adjusted groupings. *Proactive* refers to the group whose visits have regular frequency and high continuity. The optimal visit frequency of each risk-adjusted group by Centers for Medicare & Medicaid Services hierarchical condition category (CMS-HCC) score is 1, 3.46, 9.85, and 10.68, respectively (the top of each curve in A-D). Shading indicates 95% confidence intervals.

We used the ggplot2 package version 3.3.6 in R to plot and fit a generalized additive model with integrated smoothness estimation, seen as the smoothers in Figure 1 and Figure 2.^13^ We used the stats package version 3.6.3 in R to fit a multivariable regression model in eTable 3 in Supplement 1 and to perform 2-sample, 2-sided *t* tests with a significance level of .05.^14^ We used bootstrapped samples (n = 1000) to report 95% CIs for risk-adjusted outcomes. Data were analyzed from June 2022 to April 2023.

## Results

The study cohort had 504 471 Medicare fee-for-service beneficiaries, 298 422 (59.16%) were women, and the mean (SD) age was 74.26 (10.41) years. This cohort of patients had a mean 2.9 years of continuous enrollment during the baseline years. Details about the number of included beneficiaries, primary care visits, and primary care clinicians in each year during the baseline years are in eTable 2 in Supplement 1.

Descriptive data on the study cohort and subgroups are given in Table 1. A comparison of characteristics and outcomes between the 6 subgroups with different proactivity levels is given in Table 2. The mean RAF score of the subgroups decreased as the regularity and continuity of subgroups increased. Reactive care patterns were associated with greater cost and utilization. Savings increased as continuity of care increased in both regular and irregular groups, and highly continuous subgroups were the only subgroups associated with savings. Within the same continuity group, savings were higher for the regular subgroup. The difference in savings between regular and irregular subgroups of the same continuity increased as continuity decreased, which suggests that there is a tradeoff between continuity and regularity.

**Table 1.** Descriptive Data for the Study Cohort

Characteristic	All, No. (%)	By regularity and continuity subgroup, No. (%)					
		Highly continuous regular	Highly continuous irregular	Moderately continuous regular	Moderately continuous irregular	Noncontinuous regular	Noncontinuous irregular
Age, y							
22-44	9869 (1.96)	1365 (1.61)	1634 (1.81)	642 (1.59)	2059 (1.66)	681 (2.62)	3488 (2.52)
45-54	13858 (2.75)	2020 (2.38)	2160 (2.39)	1054 (2.6)	3058 (2.47)	825 (3.18)	4741 (3.42)
55-64	28 796 (5.71)	4518 (5.32)	4587 (5.07)	2290 (5.66)	6451 (5.2)	1702 (6.56)	9248 (6.67)
65-74	202 605 (40.16)	35 481 (41.79)	37 471 (41.41)	16 318 (40.3)	47 845 (38.57)	11 219 (43.23)	54 271 (39.16)
75-84	176 138 (34.92)	29 695 (34.98)	31 567 (34.89)	14 470 (35.74)	44 831 (36.14)	8488 (32.7)	47 087 (33.98)
≥85	73 205 (14.51)	11 818 (13.92)	13 063 (14.44)	5717 (14.12)	19 811 (15.97)	3039 (11.71)	19 757 (14.26)
Sex							
Male	206 049 (40.84)	38 610 (45.48)	38 310 (42.34)	17 237 (42.57)	48 834 (39.36)	10 555 (40.67)	52 503 (37.88)
Female	298 422 (59.16)	46 287 (54.52)	52 172 (57.66)	23 254 (57.43)	75 221 (60.64)	15 399 (59.33)	86 089 (62.12)
Race and ethnicity^a^							
Asian	9629 (1.91)	2056 (2.42)	2884 (3.19)	606 (1.5)	2210 (1.78)	304 (1.17)	1569 (1.13)
Black	38 459 (7.62)	8079 (9.52)	7215 (7.97)	3288 (8.12)	8293 (6.68)	2022 (7.79)	9562 (6.9)
Hispanic	6231 (1.24)	959 (1.13)	1648 (1.82)	415 (1.02)	1419 (1.14)	250 (0.96)	1540 (1.11)
North American Native	1608 (0.32)	190 (0.22)	236 (0.26)	116 (0.29)	357 (0.29)	111 (0.43)	598 (0.43)
White	431 935 (85.62)	70 034 (82.49)	74 849 (82.72)	34 775 (85.88)	108 029 (87.08)	22 489 (86.65)	121 759 (87.85)
Other/unknown	16 609 (3.29)	3579 (4.22)	3650 (4.03)	1291 (3.19)	3747 (3.02)	778 (3)	3564 (2.57)
Region							
Northeast	104 505 (20.72)	17 820 (20.99)	18 771 (20.75)	8880 (21.93)	26 289 (21.19)	5214 (20.09)	27 531 (19.86)
Midwest	105 462 (20.91)	17 819 (20.99)	16 500 (18.24)	8680 (21.44)	25 293 (20.39)	6188 (23.84)	30 982 (22.35)
South	205 847 (40.8)	35 843 (42.22)	36 666 (40.52)	17 012 (42.01)	50 860 (41)	10 398 (40.06)	55 068 (39.73)
West	87 789 (17.4)	13 286 (15.65)	18 305 (20.23)	5863 (14.48)	21 405 (17.25)	4125 (15.89)	24 805 (17.9)
Other	868 (0.17)	129 (0.15)	240 (0.27)	56 (0.14)	208 (0.17)	29 (0.11)	206 (0.15)
Disabled	49 241 (9.76)	7387 (8.7)	7838 (8.66)	3713 (9.17)	10 806 (8.71)	3001 (11.56)	16 496 (11.9)
Medicare and Medicaid dual eligibility							
Full dual	61 890 (12.27)	9864 (11.62)	11 048 (12.21)	4757 (11.75)	13 887 (11.19)	3226 (12.43)	19 108 (13.79)
Partial dual	16 520 (3.27)	2832 (3.34)	2994 (3.31)	1317 (3.25)	3729 (3.01)	956 (3.68)	4692 (3.39)
Comorbid conditions							
Cancer	38 989 (16.1)	5260 (16.92)	6210 (16.73)	3025 (16.31)	10 247 (15.89)	2002 (17.69)	12 245 (15.38)
Diabetes	46 197 (19.08)	7276 (23.4)	7192 (19.37)	4198 (22.63)	11 765 (18.25)	2118 (18.72)	13 648 (17.14)
Severe mental health disorders	21 157 (8.74)	2300 (7.4)	2864 (7.71)	1430 (7.71)	5342 (8.29)	1157 (10.23)	8064 (10.13)
Heart disorders	46 194 (19.08)	5320 (17.11)	6947 (18.71)	3259 (17.57)	12 779 (19.82)	2082 (18.4)	15 807 (19.85)
Vascular disorders	43 170 (17.83)	5613 (18.05)	6789 (18.28)	3390 (18.28)	11 632 (18.04)	1928 (17.04)	13 818 (17.36)
Chronic obstructive pulmonary disease	30 005 (12.39)	3581 (11.52)	4730 (12.74)	2207 (11.9)	8022 (12.44)	1339 (11.83)	10 126 (12.72)
Acute kidney failure	16 452 (6.79)	1740 (5.6)	2398 (6.46)	1038 (5.6)	4680 (7.26)	689 (6.09)	5907 (7.42)
RAF score, mean (range)	1.02 (0.14-15.7)	0.88 (0.14-11.89)	0.93 (0.14-10.93)	0.96 (0.14-11.91)	1.06 (0.14-12.85)	0.95 (0.14-11.3)	1.15 (0.14-15.7)

Abbreviation: RAF, risk adjustment factor.

^a^ Race and ethnicity data were obtained from the Medicare Master Beneficiary Summary File, which itself originates from Social Security Administration records.

**Table 2.** Comparison of Characteristics and Outcomes Between the 6 Groups With Different Proactivity Levels

Variable	Highly continuous regular	Highly continuous irregular	Moderately continuous regular	Moderately continuous irregular	Noncontinuous regular	Noncontinuous irregular
No. (%) of beneficiaries	84 897 (16.83)	90 482 (17.94)	40 491 (8.03)	124 055 (24.59)	25 954 (5.14)	138 592 (27.47)
No. (%) of beneficiaries with visit frequency <10.68^a^	1898 (2.24)	1743 (1.93)	1801 (4.45)	4244 (3.42)	463 (1.78)	5785 (4.17)
Visit frequency per y, median (range)	2.33 (1 to 52)	2.67 (1 to 88)	3 (1 to 79.67)	3.35 (1 to 103.56)	2 (1 to 58.77)	3.33 (1 to 83)
CoV, median (range)	0.38 (0 to 0.59)	0.82 (0.59 to 4.86)	0.47 (0 to 0.59)	0.86 (0.59 to 6.57)	0.46 (0 to 0.59)	0.92 (0.59 to 4.95)
Physician entropy, median (range)	0 (0 to 0)	0 (0 to 0)	0.05 (0 to 0.09)	0.05 (0 to 0.09)	0.12 (0.09 to 0.34)	0.13 (0.09 to 0.42)
Organizational entropy, median (range)	0 (0 to 0)	0 (0 to 0)	0.02 (0 to 0.09)	0.03 (0 to 0.09)	0.09 (0 to 0.26)	0.1 (0 to 0.42)
RAF score, mean (SD)	0.88 (0.79)	0.93 (0.85)	0.96 (0.89)	1.06 (0.99)	0.95 (0.9)	1.15 (1.08)
Medicare expenditures, mean (SD), $	8367 (19 094)	9884 (20 626)	10 451 (22 937)	12 617 (23 862)	10 791 (22 180)	14 269 (24 624)
No. of ED visits per 1000	305	391	403	509	457	640
No. of hospitalizations per 1000	159	198	199	253	213	283
Savings in Medicare expenditures, mean (SD), $	1471 (17 351)	945 (18 612)	184 (21 049)	−821 (21 531)	−647 (19 900)	−1940 (21 916)
RA Medicare expenditures (95% CI), $	10 392 (10 165 to 10 625)	11 440 (11 223 to 11 659)	11 711 (11 367 to 12 062)	12 428 (12 171 to 12 677)	12 394 (12 018 to 12 770)	12 456 (11 934 to 12 976)
RA No. of ED visits per 1000 (95% CI)	321 (314 to 328)	405 (398 to 413)	397 (387 to 406)	480 (471 to 489)	431 (418 to 445)	540 (531 to 549)
RA No. of hospitalizations per 1000 (95% CI)	166 (162 to 169)	193 (188 to 197)	184 (178 to 189)	207 (203 to 211)	198 (191 to 205)	211 (207 to 215)

Abbreviations: CoV, coefficient of variation; ED, emergency department; RA, risk-adjusted; RAF, risk adjustment factor.

^a^ 10.68 is the number of visits associated with the highest savings as seen in Figure 1.

Differences in outcomes between subgroups are summarized in Table 3. The regular and highly continuous group was associated with greater savings in Medicare expenditures (175.87%; 95% CI, 167.40% to 184.33%; *P* < .001), lower risk-adjusted Medicare expenditures (−16.61%; 95% CI, –16.73% to –16.48%; *P* < .001), fewer risk-adjusted ED visits (−40.49%; 95% CI, –40.55% to −40.43%; *P* < .001), and fewer risk-adjusted hospitalizations (−53.32%; 95% CI, –53.49% to –53.14%; *P* < .001) compared with the irregular noncontinuous group.

**Table 3.** Difference in Outcomes Between Regularity and Continuity Subgroups

Outcome	Difference between subgroups (95% CI)				
	Regular highly continuous	Irregular highly continuous	Regular moderately continuous	Irregular moderately continuous	Regular noncontinuous
**Savings in Medicare expenditures**					
Regular highly continuous	NA	NA	NA	NA	NA
Irregular highly continuous	527 (358 to 695)	NA	NA	NA	NA
*P* value	<.001				
Regular moderately continuous	1288 (1052 to 1524)	761 (523 to 999)	NA	NA	NA
*P* value	<.001	<.001			
Irregular moderately continuous	2292 (2125 to 2460)	1766 (1595 to 1936)	1004 (767 to 1242)	NA	NA
*P* value	<.001	<.001	<.001		
Regular noncontinuous	2118 (1849 to 2387)	1591 (1321 to 1862)	830 (513 to 1147)	−174 (−444 to 96)	NA
*P* value	<.001	<.001	<.001	.21	
Irregular noncontinuous	3411 (3247 to 3575)	2884 (2717 to 3052)	2123 (1888 to 2358)	1119 (952 to 1285)	1293 (1025 to 1561)
*P* value	<.001	<.001	<.001	<.001	<.001
**Risk-adjusted Medicare expenditures**					
Regular highly continuous	NA	NA	NA	NA	NA
Irregular highly continuous	−1049 (−1059 to −1039)	NA	NA	NA	NA
*P* value	<.001				
Regular moderately continuous	−1319 (−1332 to −1306)	−269 (−282 to −257)	NA	NA	NA
*P* value	<.001	<.001			
Irregular moderately continuous	−2042 (−2053 to −2032)	−993 (−1004 to −982)	−724 (−737 to −710)	NA	NA
*P* value	<.001	<.001	<.001		
Regular noncontinuous	−2005 (−2019 to −1991)	−956 (−969 to −942)	−686 (−702 to −670)	37 (23 to 52)	NA
*P* value	<.001		<.001	<.001	
Irregular noncontinuous	−2067 (−2086 to −2049)	−1018 (−1036 to −1000)	−749 (−769 to −729)	−25 (−43 to −7)	−63 (−83 to −42)
*P* value	<.001	<.001	<.001	<.001	<.001
**Risk-adjusted No. of ED visits**					
Regular highly continuous	NA	NA	NA	NA	NA
Irregular highly continuous	−84.22 (−84.55 to −83.89)	NA	NA	NA	NA
*P* value	<.001				
Regular moderately continuous	−75.35 (−75.73 to −74.98)	8.87 (8.48 to 9.26)	NA	NA	NA
*P* value	<.001	<.001			
Irregular moderately continuous	−158.79 (−159.15 to −158.43)	−74.57 (−74.94 to −74.19)	−83.44 (−83.85 to −83.02)	NA	NA
*P* value	<.001	<.001	<.001		
Regular noncontinuous	−110.23 (−110.7 to −109.76)	−26.01 (−26.49 to −25.53)	−34.88 (−35.4 to −34.36)	48.56 (48.05 to 49.06)	NA
*P* value	<.001	<.001	<.001	<.001	
Irregular noncontinuous	−218.89 (−219.25 to −218.53)	−134.67 (−135.05 to −134.29)	−143.54 (−143.96 to −143.12)	−60.1 (−60.5 to −59.7)	−108.66 (−109.16 to −108.15)
*P* value	<.001	<.001	<.001	<.001	<.001
**Risk-adjusted No. of hospitalizations**					
Regular highly continuous	NA	NA	NA	NA	NA
Irregular highly continuous	−27.05 (−27.22 to −26.87)	NA	NA	NA	NA
*P* value	<.001				
Regular moderately continuous	−18.32 (−18.53 to −18.1)	8.73 (8.51 to 8.95)	NA	NA	NA
*P* value	<.001	<.001			
Irregular moderately continuous	−41.97 (−42.15 to −41.8)	−14.93 (−15.11 to −14.75)	−23.66 (−23.87 to −23.44)	NA	NA
*P* value	<.001	<.001	<.001		
Regular noncontinuous	−32.28 (−32.54 to −32.03)	−5.24 (−5.5 to −4.98)	−13.97 (−14.25 to −13.68)	9.69 (9.43 to 9.95)	NA
*P* value	<.001	<.001	<.001	<.001	
Irregular noncontinuous	−45.04 (−45.22 to −44.87)	−18 (−18.18 to −17.82)	−26.72 (−26.94 to −26.51)	−3.07 (−3.24 to −2.9)	−12.76 (−13.02 to −12.5)
*P* value	<.001	<.001	<.001	<.001	<.001

Abbreviation: NA, not applicable.

Figure 1 adds the frequency dimension to the comparison of savings. Only the part of the graph where the confidence intervals of different groups do not overlap, ie, the part with frequency levels less than 20, was provided for clarity. When the frequency was above 20, the number of beneficiaries decreased to the point that made the difference in savings between different groups statistically insignificant. There was a concave-convex decomposition into the curves of the proactive and reactive groups. Dependence of savings in expenditures on visit frequency had a concave structure for the proactive group, with the greatest savings observed for patients receiving around 10 primary care visits annually, and the savings decreased as they deviated from the maxima of the curve. (The value 10 visits was the result of an aggregate analysis in Figure 1. The number should vary for specific patients depending on patient characteristics.) The concavity of the curve was tested by fitting a polynomial regression model with frequency and squared frequency terms. The sign of the squared term was negative (−8.72; 95% CI, –12.77 to –4.66; *P* < .001). The concave behavior of the frequency vs savings curve became convex for the reactive care group. There was a phase transition between the proactive and reactive group as regularity and continuity changed.

Optimal visit frequencies were found to increase for beneficiaries with increasing risk scores when the proactive group was divided into RAF groups, as seen in Figure 2. Curves transitioned from being downward-sloping to upward-sloping, having a concave shape during the transition as the RAF scores increased. While higher visit frequencies were associated with losses for low-risk patients, savings were maximized at more frequent visits for higher-risk patients, with 10 primary care visits per year maximizing savings only for patients with CMS-HCC scores from 1.88 to 3.1.

## Discussion

Consistent with our hypothesis, a clear association of primary care visit patterns with cost savings and utilization outcomes was observed in this large cohort derived from a national sample of Medicare fee-for-service claims. This association remained significant after adjusting for beneficiary demographics and clinical characteristics. Greater savings were observed as continuity of care increased for all patients, with highly continuous care associated with savings across all regularity and frequency groupings. Among patients who received similar continuity of care, savings were higher for those who received temporally regular visits. The greatest reductions in both risk-adjusted Medicare spending and risk-adjusted acute care utilization were observed in patients who received regular care with high continuity. The association of proactive care patterns with the highest savings is more remarkable given those patients had lower RAF scores than other subgroups. It would be more difficult to yield higher savings and utilization reductions when comparing with lower risk-adjusted benchmarks. Also, the fact that the mean RAF score of the subgroups increased as the regularity and continuity of subgroups decreased highlights the importance of risk adjustment on the outcome metrics.

For patients with proactive care patterns, as illustrated in Figure 2, the visit frequency associated with the largest difference in primary and secondary outcomes was higher for patients with greater clinical complexity as defined by the RAF score, but note that for beneficiaries with low RAF scores, higher frequency of visits was associated with deficit spending.

Cost savings were only observed for patients in the highly continuous care groups, regardless of regularity or frequency, and higher frequencies of care were only associated with more savings with the high continuity group, which suggests that continuity may be relatively more important than the other 2 factors. Across the frequency interval where the difference between the subgroups in Figure 1 was statistically significant, the regular subgroup had higher savings than its irregular counterpart within the same continuity group at any frequency. If the starting point was on the bottom curve (noncontinuous irregular), improving regularity was associated with higher savings than improving continuity, on average (across frequency levels). Increased frequency was associated with increasing the effect of improving regularity or continuity. If the starting point was on the moderately continuous, irregular curve, improving continuity was more effective than improving regularity.

All outcomes in this study, including the primary outcome, savings in Medicare expenditures, were associated with visit frequency, regularity, and continuity of care. Quantifying the magnitude of these associations is useful for suggesting the optimal characteristics of primary care practice patterns, at a beneficiary level, for health care professionals and policymakers engaged in Medicare’s transition to accountable care models. Given the interaction between these 3 characteristics, our results suggest that primary care systems and incentives under value-based care should be designed to optimize these 3 factors in combination. Proactive approaches to primary care, defined by temporally regular visits with a continuity-of-care clinician at a frequency optimized for clinical complexity, may offer benefits to payers, clinicians, and patients by decreasing expenditures, reducing ED visits, and reducing hospitalizations.

With the Association of American Medical Colleges predicting a primary care clinician shortage of between 17 800 and 48 000 clinicians by 2034,^15^ these results also highlight the importance of addressing the shortfall. Our work suggests that the supply of primary care visits available from continuity-of-care clinicians may serve as a limiting factor in Medicare’s plans to shift all traditional Medicare beneficiaries into accountable care relationships by 2030.

### Limitations

While this study was drawn from a nationally representative sample of Medicare claims, our exclusion criteria may have introduced unobserved confounders that cause higher savings for patients with higher regularity and continuity-of-care values. For example, our analysis was restricted to patients with a minimum of 3 primary care visits, and thus we are limited in our ability to evaluate the benefits of primary care visit patterns for patients not currently accessing primary care.

Our results do not establish causality. This is because there may be unobserved patient- or clinician-level variables that we cannot capture during the risk-adjustment process. For example, we do not know whether the patients in the reactive group have irregular and noncontinuous visit patterns because their primary care clinicians are not trying to stabilize their visit patterns or because the patients are not responsive to their primary care clinicians’ attempts to stabilize their visit patterns. Thus, it is unknown whether moving a patient from reactive to a proactive care model by altering their visit patterns would improve outcomes and give rise to a concave shape in their frequency vs savings curve. In future research, a random sample of patients receiving irregular and noncontinuous care could be enforced to have regular and continuous visit patterns by their primary care clinicians. The difference between the outcomes and the shape of the frequency vs savings group could then be compared between those who are responsive to the enforcement and those who are not.

## Conclusions

In this cohort study of 504 471 Medicare fee-for-service beneficiaries, we found that savings in Medicare expenditures and risk-adjusted values for Medicare expenditures and number of ED visits and hospitalizations were associated with visit frequency, regularity, and continuity in primary care. Optimization of these primary care visit patterns was associated with significant improvement in outcomes. Quantifying these associations and demonstrating the interdependency of these associations is useful for health care professionals and policymakers as Medicare continues its transition to value-based reimbursement models.

## Notes

Supplement 1. **eTable 1.** Primary care CPT codes used to identify primary care visits **eTable 2.** Number of included beneficiaries, PC visits and PCPs by years **eTable 3.** Regression table with the dependent variable savings in Medicare expenditures

Supplement 2.
eMethods

eReferences
**eFigure 1.** Flow chart of numbers of beneficiaries at each stage of study **eFigure 2.** A visualization of how the continuity of care cutoffs are applied

Supplement 3.
Data Sharing Statement
