# Supplementary material for: Primary Care Continuity, Frequency, and Regularity Associated With Medicare Savings
Source: JAMA Netw Open. 2023 Aug 21;6(8):e2329991. doi: 10.1001/jamanetworkopen.2023.29991 (PMC10442711; doi:10.1001/jamanetworkopen.2023.29991)
Supplement: Supplement 2. — eMethods eReferences eFigure 1. Flow chart of numbers of beneficiaries at each stage of study eFigure 2. A visualization of how the continuity of care cutoffs are applied [file jamanetwopen-e2329991-s002.pdf]

Supplemental Online Content

Sonmez D, Weyer G, Adelman D. Primary care continuity, frequency, and regularity associated with Medicare savings. *JAMA Netw Open*. 2023;6(8):e2329991. doi:10.1001/jamanetworkopen.2023.29991

eMethods

eReferences

eFigure 1. Flow chart of numbers of beneficiaries at each stage of study

eFigure 2. A visualization of how the continuity of care cutoffs are applied

This supplemental material has been provided by the authors to give readers additional information about their work.

© 2023 Sonmez D et al. *JAMA Network Open.*

eMethods

Sample selection

Beneficiaries who had '0' (not terminated) listed as their part A termination code and part B termination code, who had a null date of death, who had '3'(Parts A and B) or 'C'(Parts A and B state buy-in) listed as their monthly Part A and/or Part B entitlement indicator for all months of the file year, who didn't have '2'(ESRD) or '3' (both disability insurance benefits and ESRD) as their current reason for entitlement, who didn't have '11'(aged with ESRD), '21'(disabled with ESRD) or '31'(ESRD only) as their Medicare status code during any month of the file year, who had '0'(not a member of a MA plan) listed as their monthly MA enrollment indicator for all months of the file year in all MBSF base files corresponding to the observation and outcome years, were filtered to identify beneficiaries who were continuously enrolled in Medicare Parts A and B, who were not enrolled in Medicare Advantage (MA), and who did not have end-stage renal disease (ESRD) during the observation years and the outcome year. Patients who satisfy these criteria are called eligible, and the first year they start satisfying these criteria is called their first year of eligibility. Suppose a beneficiary starts satisfying these criteria during 2017, 2018, or 2019. In that case, they are still considered eligible for the study cohort. However, their visits before their first year of eligibility are discarded while calculating the predictive measures.

We excluded patients who had at least one primary care visit documented in outpatient claims without any match in the carrier claims. Due to the incomplete linkages between these different data sources, the billing provider in outpatient claims is unknown unless a matching claim can be found in the carrier claims, and the billing provider is necessary while calculating organizational entropy of a beneficiary. Participant flow is summarized in eFigure 1.

Identifying PC visits

PC visits were identified using the outpatient and carrier claim files to identify such patients. If a carrier or an outpatient claim has at least one of the Healthcare Common Procedure Coding System (HCPCS) codes listed in eTable1 associated with it and if the performing physician has a primary taxonomy code of a primary care physician (i.e., has a primary taxonomy code corresponding to the titles internal medicine, general practice, family medicine, nurse practitioner, physician assistant or geriatrics), that claim is labeled as a PC claim.

© 2023 Sonmez D et al. *JAMA Network Open.*

Matching Outpatient and Carrier Claims to Prevent Double Counting of Visits

Since a patient can have a claim in both the outpatient claims file and the carrier claims file associated with the same visit, claims in these two files had to be matched to prevent the double-counting of visits. The match was performed by enforcing a match between the beneficiary ID and the claim date. The condition that at least one physician's national provider identification number (NPI) be listed in both claims was also enforced.

An NPI is a unique identification number assigned to healthcare providers. There are many types of providers associated with a claim. In the outpatient claims base file, there are attending physician, operating physician, rendering physician, referring physician, other physician, service location, and organization NPI columns that are not necessarily populated for all claims. The outpatient claims revenue file has a rendering physician NPI column. Since multiple lines are associated with one claim in the revenue files, there can be multiple rendering physician NPIs associated with one claim. Similarly, there are referring physician and billing organization NPI columns in the carrier base file. There are performing physician and organization NPI columns in the carrier claims revenue file. While matching the claims across the outpatient and carrier files, the condition that at least one of the physician NPIs listed in the attending, operating, rendering or other physician NPIs of the outpatient claim should match at least one of the performing physician NPIs of the carrier claim, was enforced.

CMS's Expected Medicare Expenditures Calculation

Using CMS's method, expected Medicare expenditures are calculated as follows:

RAF score × Monthly Benchmark12 × Normalization Factor

The terms given in this formula are explained below.

Risk adjustment factor (RAF) score

A RAF score is the sum of a beneficiary's hierarchical condition categories (HCC) score and the demographic relevant factor. Demographic relevant factors depend on sex, age, the reason for Medicare eligibility, institutional status, and dual eligibility status of a beneficiary. Possible reasons for Medicare eligibility are old age, having a disability, or having ESRD. If a beneficiary is a long-term resident in an institution, primarily a nursing facility, the beneficiary is said to have an institutional residence. [1] This study identified beneficiaries with a skilled nursing facility claim that lasted for at least 180 days prior to the outcome year as institutionalized patients. Beneficiaries enrolled in Medicare Part A or Part B and receiving Medicaid benefits or assistance with Medicare premiums are

© 2023 Sonmez D et al. *JAMA Network Open.*

called dual eligibles.[1] CMS annually publishes demographic relevant factors for each subpopulation with different ages, sex, reason for Medicare eligibility, institutional status, and dual eligibility status.[2]

HCC Score is the second component of the RAF score. It accounts for the beneficiary's health status while determining how much the beneficiary's expected Medicare expenditure differs from an average Medicare beneficiary. It considers the presence of certain diagnosis codes in the current year to predict the expected costs in the next year. CMS publishes the regression models used to calculate HCC scores. These models are updated yearly to account for temporal changes such as changes in diagnosis coding practices and to improve the explanatory and predictive performance of the model. The most recent version of the CMS's risk adjustment model, the CMS-HCC model, was used to predict the costs in 2019. This model version was designed to have high predictive power for subpopulations. The subpopulations described above are segments of the Medicare population with common sex, age, reason for Medicare eligibility, institutional status, and dual eligibility status. Separate regression models were trained for subpopulations, and the relevant model was employed given the input characteristics of the patients. The model also inputs ICD-9 or ICD-10 codes and maps them to approximately 1500 diagnostic groups. Each diagnostic group represents a well-specified condition. The diagnostic group codes are further classified into 204 condition categories (CCs). Each CC includes conditions that are medically similar and that have comparable costs. Hierarchies are imposed on CCs to obtain 86 hierarchical condition categories (HCCs). Hierarchies ensure that only the most severe conditions among related conditions are used to calculate the HCC comorbidity score. Finally, the model determines the subpopulation each beneficiary belongs and uses the HCC conditions obtained from the input diagnostic codes to predict the HCC score.[1]

Benchmarks

CMS publishes yearly benchmarks, also called Medicare Advantage rates, for each county.[3] Benchmarks are the expected cost of an average beneficiary in Original Medicare in the payment year. Therefore, when multiplied by the proper risk score, the expected cost of the beneficiary in the payment year can be obtained. As our evaluation was conducted on a fee for service population, we chose not to use the benchmarks (payment rates) published by CMS for Medicare Advantage patient populations as these payment rates are created for population of patients receiving managed care. In our case, a geographically adjusted cost benchmark derived from our fee for service Medicare data in was thought to provide a more accurate comparison. To account for the structural difference between Medicare Advantage and Fee-For-Service systems, the average Medicare expenditures of the population were calculated for

© 2023 Sonmez D et al. *JAMA Network Open.*

each county and these values were used instead of the benchmarks published by CMS. We used the average expenditures of all beneficiaries in our data in 2019.

Normalization factor

CMS-HCC score model was calibrated using the 2014-2015 data. Therefore, when this model is used to predict the Medicare expenditures in a year other than 2015, the average RAF score among all beneficiaries in that year may not normalize to 1. The average RAF score among all beneficiaries should be 1.0 so that RAF scores align with the benchmarks by representing the degree to which a beneficiary's health status results in expected costs that are more or less than the expected cost of the average beneficiary. As a remedy, CMS publishes a normalization factor to be multiplied by RAF scores every year. The relevant normalization factor for this study was announced to be 1.038.[2]

Identifying ED Visits

Outpatient claims with revenue center codes 0450-0459 or 0981 were extracted so that ED visits that didn't lead to a hospitalization can be identified. Claims with HCPCS codes 70000-79999 or 80000-89999 were excluded since the presence of these codes means that only radiological or pathology/laboratory services were provided

Identifying Hospitalizations

Inpatient claims that have 0001-0879 or 1300-1399 as the last 4 digits of their provider numbers were extracted so that inpatient claims of traditional acute care hospitals or critical access hospitals can be identified. The number of claims with distinct provider numbers and admission dates were counted for each beneficiary to prevent double counting of hospital stays that generated multiple facility claims.[4]

© 2023 Sonmez D et al. *JAMA Network Open.*

eReferences

1. Centers for Medicare and Medicaid Services. Report to congress: Risk adjustment in medicare advantage – December 2021. https://www.cms.gov/Medicare/Health-Plans/MedicareAdvtgSpecRateStats/Risk-Adjustors-Items/ReportToCongress. Accessed June 6, 2022.
2. Centers for Medicare and Medicaid Services. Announcement of calendar year (cy) 2019 medicare advantage capitation rates and medicare advantage and part d payment policies and final call letter. https://www.cms.gov/Medicare/Health-Plans/MedicareAdvtgSpecRateStats/Announcements-and-Documents. Accessed June 6, 2022.
3. Centers for Medicare and Medicaid Services. Medicare Advantage Rates & Statistics. https://www.cms.gov/Medicare/Health-Plans/MedicareAdvtgSpecRateStats. Accessed June 6, 2022.
4. Research Data Assistance Center. Medicare Inpatient File, LDS or RIF: Counting Distinct Hospitalizations. https://resdac.org/articles/medicare-inpatient-file-lds-or-rif-counting-distinct-hospitalizations. Accessed June 6, 2022.

© 2023 Sonmez D et al. *JAMA Network Open.*

eFigure 1. Flow chart of numbers of beneficiaries at each stage of study

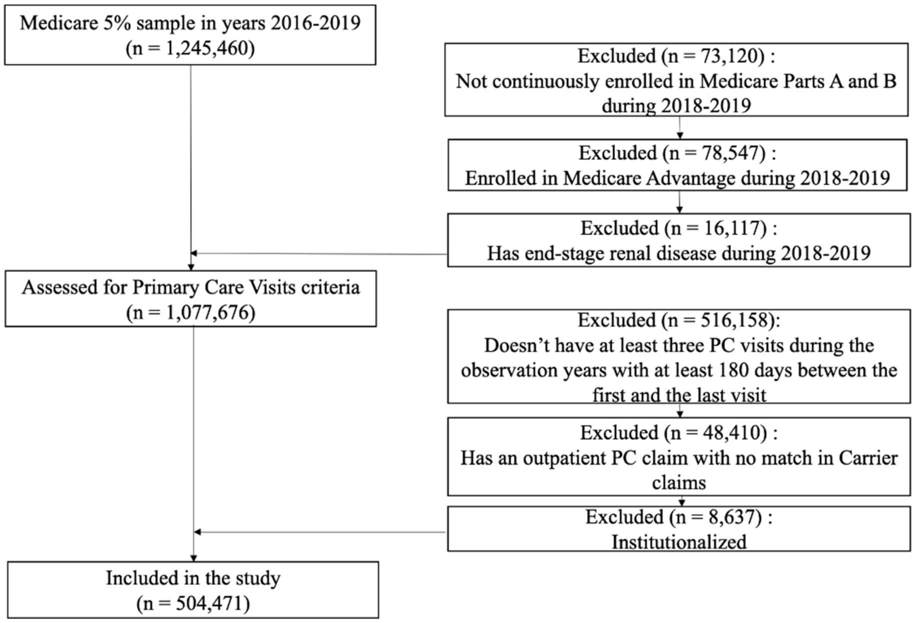

eFigure 2. A visualization of how the continuity of care cutoffs are applied

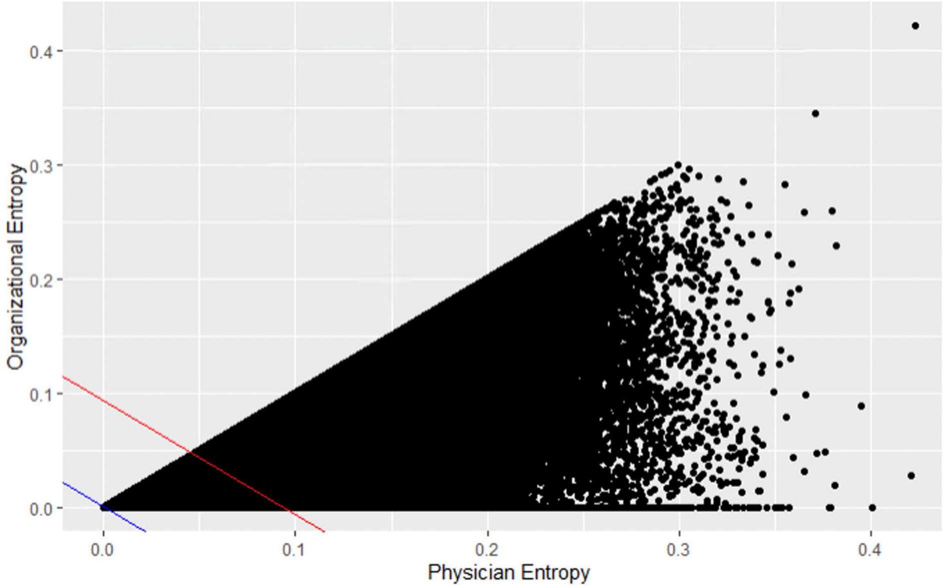

Beneficiaries on the blue line are highly continuous, beneficiaries in between the blue and red line or on the red line are moderately continuous, and the beneficiaries above the red line are noncontinuous.

© 2023 Sonmez D et al. *JAMA Network Open.*
