# Supplementary material for: Primary Care Continuity, Frequency, and Regularity Associated With Medicare Savings
Source: JAMA Netw Open. 2023 Aug 21;6(8):e2329991. doi: 10.1001/jamanetworkopen.2023.29991 (PMC10442711; doi:10.1001/jamanetworkopen.2023.29991)
Supplement: Supplement 3. — Data Sharing Statement [file jamanetwopen-e2329991-s003.pdf]

Data Sharing Statement

Sonmez. Primary Care Continuity, Frequency, and Regularity Associated With Medicare Savings. *JAMA Netw Open*. Published August 21, 2023.
doi:10.1001/jamanetworkopen.2023.29991

Data
Data available: Yes
Data types: Deidentified participant data, Data dictionary
How to access data: https://www.cms.gov/research-statistics-data-and-systems/files-for-order/limiteddatasets
When available: With publication

Supporting Documents
Document types: None

Additional Information
Who can access the data: researchers as allowed by federal laws and regulations as well as CMS policy.
Types of analyses: for a specified purpose
Mechanisms of data availability: with a signed data access agreement
